# Reduction of higher-order occipital GABA and impaired visual perception in acute major depressive disorder

**DOI:** 10.1038/s41380-021-01090-5

**Published:** 2021-04-16

**Authors:** Xue Mei Song, Xi-Wen Hu, Zhe Li, Yuan Gao, Xuan Ju, Dong-Yu Liu, Qian-Nan Wang, Chuang Xue, Yong-Chun Cai, Ruiliang Bai, Zhong-Lin Tan, Georg Northoff

**Affiliations:** 1 Affilianted Mental Health Center & Hangzhou Seventh People’s Hospital, Interdisciplinary Institute of Neuroscience and Technology, Zhejiang University School of Medicine, Hangzhou, China; 2 Key Laboratory of Biomedical Engineering of Ministry of Education, Qiushi Academy for Advanced Studies, College of Biomedical Engineering and Instrument Science, Zhejiang University, Hangzhou, China; 3 Department of Psychology and Behavioral Sciences, Zhejiang University, Hangzhou, China; 4 University of Ottawa Institute of Mental Health Research and University of Ottawa Brain and Mind Research Institute, Ottawa, ON Canada

**Keywords:** Biochemistry, Depression

## Abstract

Major depressive disorder (MDD) is a complex state-dependent psychiatric illness for which biomarkers linking psychophysical, biochemical, and psychopathological changes remain yet elusive, though. Earlier studies demonstrate reduced GABA in lower-order occipital cortex in acute MDD leaving open its validity and significance for higher-order visual perception, though. The goal of our study is to fill that gap by combining psychophysical investigation of visual perception with measurement of GABA concentration in middle temporal visual area (hMT+) in acute depressed MDD. Psychophysically, we observe a highly specific deficit in visual surround motion suppression in a large sample of acute MDD subjects which, importantly, correlates with symptom severity. Both visual deficit and its relation to symptom severity are replicated in the smaller MDD sample that received MRS. Using high-field 7T proton Magnetic resonance spectroscopy (^1^H-MRS), acute MDD subjects exhibit decreased GABA concentration in visual MT+ which, unlike in healthy subjects, no longer correlates with their visual motion performance, i.e., impaired SI. In sum, our combined psychophysical-biochemical study demonstrates an important role of reduced occipital GABA for altered visual perception and psychopathological symptoms in acute MDD. Bridging the gap from the biochemical level of occipital GABA over visual-perceptual changes to psychopathological symptoms, our findings point to the importance of the occipital cortex in acute depressed MDD including its role as candidate biomarker.

## Introduction

Major depressive disorder (MDD) is a complex, usually recurring psychiatric illness characterized by pervasive disturbances, such as mood dysregulation, impaired cognitive control and behavior [1]. Biomarkers linking psychophysical and biochemical changes in acute depressed MDD remain elusive so far, though.

MDD exhibits wide-spread cortical changes in different networks including the occipital cortex that shows reduced activity [2–4] and therapeutic responsiveness to transcranial magnetic stimulation [5]. Earlier studies using magnetic resonance spectroscopy (MRS) repeatedly show reduction in inhibitory GABA in occipital cortex of MDD [6, 7 see 8, though]. Involvement of inhibitory GABA in occipital cortex must also be distinguished from the frontal cortex in MDD where changes in excitatory glutamate/glutamine seem to predominate [9, 10]. Moreover, occipital GABA levels in MDD are state-dependent [11] as they show “normalization” after successful electroconvulsive [12], cognitive behavioral therapy [13] and psychopharmacological treatment with selective serotonin reuptake inhibitors (SSRI [14]). Together, these studies suggest occipital GABA to be a promising candidate biomarker of the acute depressed state. However, these findings leave open whether occipital GABA is directly related to psychophysical impairment in visual perception and ultimately to psychopathological symptoms of MDD—this would further strengthen confidence in both its validity [8] and utility as biomarker of the acute depressed state. Filling this gap in our knowledge by combining psychophysical investigation of visual perception with biochemical measurement of occipital GABA in acute depressed MDD is the main aim of our study.

A well-known visual paradigm, motion center-surround interactions, can evoke motion spatial suppression in high contrast moving gratings [15, 16]. This paradigm reflects visual motion processing in human higher-order occipital-middle temporal area (MT) [15–17], and has been applied to the study of multiple clinical phenomena including schizophrenia [18], autism [19, 20], and MDD [21]. Observing disease-specific findings, Golomb et al. [21] reported that remitted MDD patients show decreased motion suppression in high contrast stimuli which, as they propose, may be associated with GABA deficits in occipital cortex (early visual cortex, EVC). Are deficits in visual perception, i.e., motion suppression related to occipital GABA-decrease in acute depressed MDD? The goal of our study is to fill this gap in our current knowledge by combining psychophysical investigation of visual perception with biochemical measurement of occipital cortex GABA in the same MDD subjects.

We hypothesize that acute MDD subjects show a deficit in specifically surround motion suppression (as distinguished from, for instance, duration threshold). Moreover, we hypothesize such specific visual deficit to be related to decreases in GABA concentration in occipital cortex. We focus on a specific subarea on the lateral occipital sulcus- MT+ [22] known to be involved in motion discrimination performance and to be modulated by inhibitory GABA [16]. Therefore, we measure GABA and glutamate concentrations in human MT complex (hMT+) using ^1^H-MRS at ultra-high-field 7T, and compared both metabolites between acute depressed MDD and healthy subjects.

We first demonstrate a highly specific psychophysical impairment in visual perception in a larger sample of acute MDD including its relation to symptom severity. Both visual deficit and its relation to symptom severity are replicated in the smaller MDD sample that underwent MRS. Secondly, we show decreased GABA concentration in higher-order visual cortex, i.e., hMT+ in acute depressed MDD subjects which correlates with their psychophysical deficit in visual perception. Together, we demonstrate occipital GABA deficit in acute MDD and impaired visual perception with the latter relating to symptom severity. Bridging the gap from biochemical over psychophysical to psychopathological levels, our findings point to the importance of the occipital cortex in MDD and its role as candidate biomarker and treatment target [5].

## Materials and methods

### Participants

This work was approved by the Ethics committee of Hangzhou Seventh People’s Hospital. All participants had written the informed consents. We performed two studies. Study 1: 70 adult individuals with acute MDD patients, and 52 normal controls (Supplementary Table 1) participated in the behavioral experiment of spatial suppression psychophysically. Study 2: 18 adult MDD subjects, and 20 healthy subjects with age- and gender-matched (Supplementary Table 2), participated in the spatial suppression psychophysical and MRS experiments. All subjects had normal or corrected to normal vision. Inclusion and exclusion criteria are described in Supplementary method.

### Measurement of motion spatial suppression

All stimuli were generated using Matlab (MathWorks, Natick, MA) with Psychophysics Toolbox [23]. Study 1 (a large sample of participants) was performed in hospital, we used the portable computer (ROG 3) to conduct the motion suppression experiment. Study 2 (smaller sample) was performed in the 7T imaging center, visual stimuli were shown on a linearized monitor (Cambridge Research System, UK). For details of the procedure for measurement are presented in  Supplementary method. Briefly, a schematic of the stimuli and trial sequences is shown in Supplementary Fig. 1 (Psychophysical Task). The stimulus was a vertical drifting sinusoidal grating (contrast: 50%, high contrast); spatial frequency, 1 cycle/°; speed, 4°/s) of either small (diameter of 2°) or large (diameter of 10°) size. The edge of the grating was blurred with a raised cosine function (width, 0.3°). The grating was ramped on and off with a Gaussian temporal envelope, and the grating duration was defined as 1 SD of the Gaussian function. The duration was adaptively adjusted in each trial, and duration thresholds were estimated by a staircase procedure. Thresholds for large and small gratings were obtained from a 160-trial block that contained four interleaved 3-down/1-up staircases. For each participant, we computed the correct rate for different stimulus durations separately for each stimulus size. These values were then fitted to a cumulative Gaussian function, and the duration threshold corresponding to the 75% correct point on the psychometric function was estimated for each stimulus size. To quantify the spatial suppression strength, we calculated the spatial suppression index (SI), defined as the difference of log10 thresholds for large versus small stimuli [15, 16].

1 $${\mathrm{SI}} = \log _{10}\left( {\mathrm{{large}}\;{\mathrm{threshold}}} \right) - \log _{10}\left( {\mathrm{{small}}\;{\mathrm{threshold}}} \right)$$

### MR experimental procedure

MR experiments were performed in a 7T whole body MR system (Siemens Healthcare, Erlangen, Germany) with a Nova Medical 32 channel array head coil. MRS data were collected within hMT+ for each subject. Session included structural image scanning, fMRI localizer scan (part of the subjects), and MRS scan for the hMT+. Structural image scans were acquired with a 0.7 mm isotropic resolution MP2RAGE sequence (TR/TI1/TI2 = 5000/901/3200 ms). Spectroscopy data were acquired using a ^1^H-MRS single-voxel short-TE STEAM (Stimulated Echo Acquisition Mode) sequence [24] (TE/TM/TR = 6/32/7100 ms) with 4096 sampling points, 4-kHz bandwidth, 16 averages, 8 repetitions, 20 × 20 × 20 mm^3^ VOI size, and VAPOR (variable power and optimized relaxation delays) water suppression [25]. Prior to acquisition, first- and second-order shims were adjusted using FASTMAP (fast, automatic shimming technique by mapping along projections) [26]. Two non-suppressed water spectra were also acquired: one for phase and eddy current correction (only RF pulse, 4 averages) and another for metabolite quantification (VAPOR none, 4 averages). Voxels were positioned based on anatomical landmarks using a structural image scan collected in the same session, while avoiding contamination by CSF, bone, and fat. The procedure of locating the hMT+ VOIs were described in Supplementary method.

### MRS data processing

For details of the procedure for estimating the metabolite concentrations were displayed in Supplementary method. Briefly, spectroscopy data were preprocessed and quantified using MRspa (magnetic resonance signal processing and analysis, https://www.cmrr. umn.edu/downloads/mrspa/), which runs under MATLAB and invokes the interface of the LCModel (Version 6.3-1 L) [27]. Our concentrations were mM per kg wet weight. Furthermore, LCModel analysis was performed on all spectra within the chemical shift range of 0.2–4.0 ppm.

The signal-to-noise ratio (SNR) and full-width at half maximum (FWHM, the estimate of linewidth) were used to control the spectral quality. All spectra with SNR < 15 or FWHM > 18 Hz were discarded. Metabolite concentrations with Cramer-Rao Lower Bound (CRLB) > 20% were also excluded from further analysis.

### Data analysis

SPSS (version 20.0) was used to perform all statistical analysis in this study. To analyze the correlation, we used Pearson’s correlation coefficient. Student’s *t* test (mean ± SD) was used to determine difference between groups. The general linear regression analysis method is used to analyze the relationships. Differences were considered statistically significant if *p* < 0.05. In cases where multiple comparisons were made, false discovery rate correction was used to adjust *p* value.

## Results

### From visual perception to symptoms—Motion spatial suppression in MDD and HC

Motivated by the previous study showing reduced motion suppression in remitted MDD [21], we conducted the spatial suppression psychophysical experiment in two samples of participants, one larger sample with 70 MDD and 52 HC subjects, the other smaller one with 18 MDD and 20 HC subjects. The two psychophysical experiments were performed in different locations using different measurement devices (see materials and methods). The larger sample served to establish the psychophysical changes in visual perception while the smaller sample was used to replicate the former. Moreover, unlike the larger sample, the smaller sample also underwent MRS.

To quantify spatial suppression psychophysically, we measured duration thresholds for discriminating the motion direction of sinusoidal gratings of small and large stimuli (Fig. S1). Due to surround suppression, it was more difficult for subjects to judge the motion direction of a large grating than a small one. Therefore, we used the difference in duration thresholds between the large and small size stimuli to evaluate the strength of surround suppression (i.e., spatial SI; see materials and methods).

**Fig. 1:**
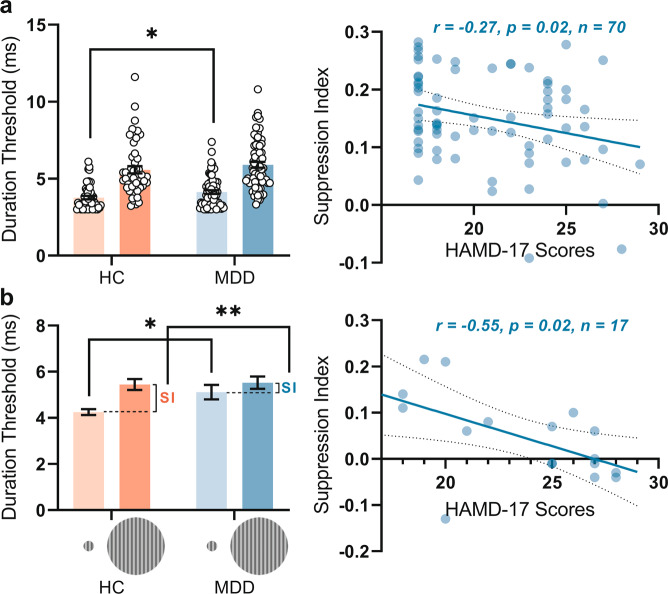
The results of behavioral experiment in MDD and HC groups in a large (a) and smaller (b) samples. **a** The results of a large sample. Left: The mean value of duration thresholds of small stimuli is significantly higher in acute MDD (4.13 ± 0.98 ms, *n* = 70) than HC group (3.77 ± 0.81 ms, *n* = 52) (*p* = 0.03). There is no significant difference of mean thresholds in large stimuli between acute MDD (5.90 ± 1.61 ms, *n* = 70) and HC groups (5.57 ± 1.70 ms, *n* = 52) (*p* = 0.29). Right: there is significant negative correlation between motion SI and HAMD scores. **b** The results of the smaller sample. Left: The mean duration thresholds of small stimuli is significantly higher in acute MDD (5.11 ± 1.28 ms, *n* = 17) than HC group (4.25 ± 0.58 ms, *n* = 20) (*p* = 0.01). There is no significant difference of mean thresholds in large stimuli between acute MDD (5.52 ± 1.11 ms, *n* = 17) and HC groups (5.45 ± 1.07 ms, *n* = 20) (*p* = 0.83). Right: there is significant negative correlation between motion SI and HAMD scores. Error bars in (**a** and **b**)**:** ±SEM.

In our large sample, we observed that MDD subjects show lower SI (not significant, though) when compared to healthy subjects. We found there was a significant differences of small stimuli’s duration threshold between MDD and HC groups, whereas no significant differences in the large stimuli’s duration threshold between the two groups (Fig. 1a left). Moreover, we found that the motion SI significantly correlated negatively with symptom severity as measured by HAMD: the smaller the SI in visual perception, the higher the HAMD-score indicating higher degrees of symptom severity (Fig. 1a right).

Both psychophysical findings, significantly increased duration threshold in specifically small stimuli (not in large stimuli) and motion SI showing negative correlation with symptom severity were replicated in the small MDD sample (see Fig. 1 b). Unlike in the larger MDD group where SI was lower but not significantly so, we found that in the smaller sample, the acute depressed MDD subjects showed a significantly (*p* = 0.007) lower SI (0.05 ± 0.09, *n* = 17) than the control group (0.10 ± 0.06, *n* = 20) (Fig. 1b left). Together, we show decrease in SI in MDD within both large and small samples; however, that SI decrease was significant only in the small sample but not in the large sample. This, as we assume, may be related to either differences in the MDD samples and/or the different display apparatus which may have affected especially the presentation of the small stimuli. Finally, it shall be mentioned that decreased SI correlated negatively with symptom severity (HAMD) in both large and small MDD samples (Fig. 1a and b right).

### Biochemical findings—Measuring GABA and glutamate concentrations in 7T MRS

**Fig. 2:**
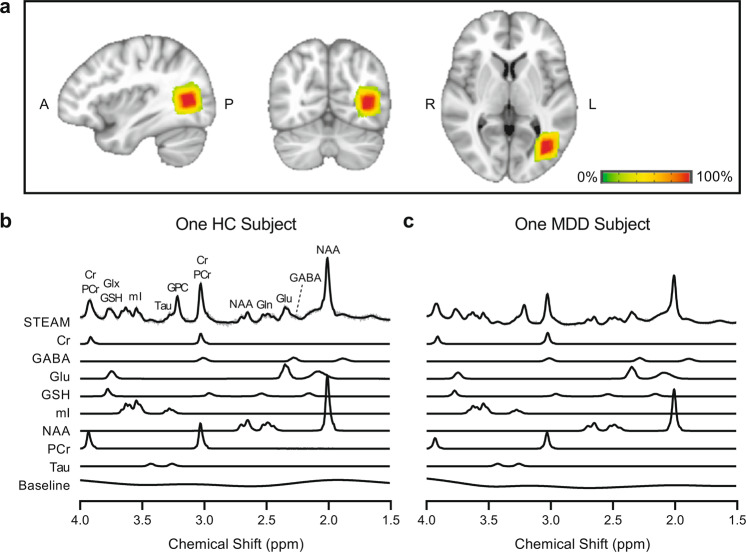
Average voxel placements (hMT+) and LCModel fit examples. **a** MRS voxels for hMT+. Green-red color indicates the percent overlap of the MRS Voxels in the hMT+ region (in MNI 152 space) across all subjects in the MDD and HC groups. From left to right are sagittal, coronal, and horizontal views. As a check on voxel placement, for ten control participants, we acquired functional localizers for hMT+ using fMRI. The MDD group were totally anatomically identified. We only used the left hMT+ as the target region (see Supplementary method). **b** and **c** Examples of metabolite spectra and LCModel fit the examples. Spectrum examples were from a control subject (**b**) and a depression subject (**c**). The first line is the LCModel fitting result of total metabolites, and the following lines show the decomposition spectrum of different metabolites.

^1^H-MRS was acquired at 7T within a specific volume of interest (VOI) in hMT+ with a size of 20 × 20 × 20 mm^3^ in our smaller acute depressed MDD sample. The hMT+ VOIs were identified by anatomical landmarks [16, 28]. The MRS hMT+ voxels were averaged across all subjects in MNI 152 space, which is shown in Fig. 2 a. Example spectra of two participants (one HC subject, the other MDD patient) are shown in Fig. 2 b and c.

**Fig. 3:**
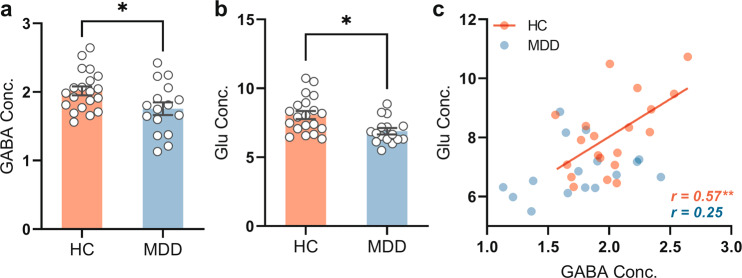
Comparison of the GABA and Glu concentrations in MDD and HC groups. **a** Comparison the mean GABA concentration in MDD (*n* = 16) and HC group (*n* = 20). **b** Comparison the mean Glu concentration in MDD (*n* = 16) and HC group (*n* = 20). Error bars in (**a** and **b**)**:** ±SEM. *p < 0.05. **c** There is significant positive correlation between GABA and Glu in HC group (*r* = 0.57, *p* = 0.008, *n* = 20). There is no significant correlation between GABA and Glu in acute MDD group (*r* = 0.25, *p* = 0.34, *n* = 16). GABA and Glu concentrations (Conc.) are absolute, with units of mmol/kg wet weight (for details see materials and methods).

MRS measures of GABA levels were analyzed for the effect of diagnosis. As shown in Fig. 3 a, the GABA concentration was significantly (*p* < 0.05) lower in MDD subjects (1.76 ± 0.37 mmol/kg, *n* = 16) than in healthy control subjects (2.02 ± 0.29 mmol/kg, *n* = 20). Glu concentrations were also significantly (*p* = 0.02) lower in MDD (6.89 ± 0.91 mmol/kg, *n* = 16) than in HC (8.07 ± 1.3 mmol/kg, *n* = 20) (Fig. 3 b).

There was significantly (*r* = 0.57, *p* = 0.008, *n* = 20) positive correlation between GABA and glutamate levels in the HC group (Fig. 3 c), reflecting excitation-inhibition balance (EIB) [29]. The EIB may reflect the neural mechanism that underlies normalization processing, which is believed to contribute to canonical neural computation [30] as key in motion spatial suppression in hMT+ [16]. In contrast to HC, there was no significant relationship of GABA with glutamate levels in MDD (*r* = 0.25, *p* = 0.34, *n* = 16) (Fig. 3 c). Albeit tentatively, this suggests abnormal EIB with decoupling of inhibitory GABA from excitatory glutamate. Other metabolites are described in Supplementary results (including Supplementary Fig. 2 and Supplementary Table 3).

### From biochemistry to visual perception and psychopathology—disrupted relation of GABA and Glu to SI in MDD

In HC group, we found that SI correlated well with both GABA concentration (*r* = 0.49, *p* = 0.03, *n* = 20) and Glu concentration (*r* = 0.53, *p* = 0.02, *n* = 20), such that HC participants with higher GABA and Glu concentrations displayed stronger motion spatial suppression. We also found that there was significant correlation between Glu and GABA (*r* = 0.57, *p* = 0.008, *n* = 20) in hMT+ in healthy subjects. This allowed us to conduct hierarchical regression model with SI as dependent variable while GABA and Glu concentrations served as predictors.

**Fig. 4:**
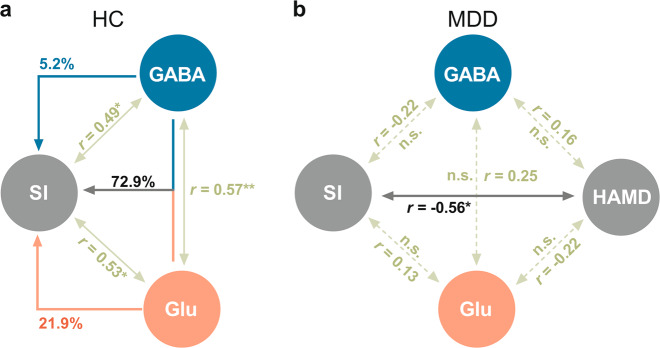
Summary and detailed analyze the influences of GABA and Glu concentrations on the behavioral scores (SI and HAMD). **a** Summary the significant correlations among SI, GABA and Glu concentrations in HC group. Furthermore, hierarchical regression model presents that the SI explained by the concentrations of GABA and Glu: 72.9% of SI variance was explained by conjoint GABA and Glu concentrations, 21.9% was uniquely explained by Glu concentration alone, and 5.2% by GABA concentration alone. **b** Summary the correlations among SI, HAMD, GABA and Glu concentrations in MDD group. There is only a significant negative correlation between motion SI and HAMD, no other significant correlations among the four variables.

The variance of SI explained by the concentrations of GABA and Glu showed the following results in HC: 72.9% of SI variance was explained by conjoint GABA and Glu concentrations, 21.9% was uniquely explained by Glu concentration alone, and 5.2% by GABA concentration alone (see Fig. 4 a). We obtained a contrasting picture in MDD. Unlike in the HC, impaired SI in acute MDD neither correlates with GABA (*r* = −0.22, *p* = 0.43, *n* = 16) nor with Glu (*r* = 0.13, *p* = 0.65, *n* = 16) concentrations; this rendered impossible to conduct a hierarchical regression model in MDD as to analyze the detailed influences of GABA and Glu on SI as we did in HC (see Fig. 4 b).

Since the hierarchical regression model could not be applied in MDD, we instead calculated moderation model to analyze the influences of GABA or Glu levels on visual perception (SI) and symptom severity (HAMD). We used the independent variable(X)-SI, moderation variable (M)-Glu/or GABA, and the interactive term of SI and Glu/or GABA to explain the dependent variable (Y) namely HAMD (shown in Supplementary Fig. 3). The results show that neither GABA nor Glu exert a significant regulatory effect on the dependent variable, i.e., HAMD, through visual perception, i.e., SI. (shown in Supplementary Table 4).

Together, these results demonstrate that the coupling of GABA and Glu to visual perception (SI), as established in healthy subjects in our data, is no longer present in MDD subjects. Our findings suggest that the relation of reduced GABA (and Glu) to impaired visual perception (SI) is disrupted in acute MDD. While the degree of that biochemical-psychophysical disruption is related to the psychopathological level, namely symptom severity.

## Discussion

Conducting a combined psychophysical-behavioral study, we demonstrate a specific impairment in visual perception, i.e., surround motion suppression, including its relation to symptom severity in a large sample of acute MDD subjects. This is replicated in a smaller MDD sample that, additionally, shows decreased GABA concentration in higher-order occipital cortex, i.e., hMT+ with the latter relating to the specific visual perception deficit. Together, we link biochemical, psychophysical, and psychopathological levels in our investigation of the occipital cortex in acute MDD. Albeit preliminarily due to the lower number of MRS subjects, our findings hint toward a key role of occipital inhibitory GABA in mediating specific higher-order visual impairment in acute depressed MDD. Together with previous data, this marks occipital GABA a suitable candidate biomarker, i.e., endophenotypic marker [31], of acute depressed MDD.

### Reduced visual motion suppression in acute depressed MDD

A previous study by Golomb et al [21]. reported that remitted MDD subjects exhibit decreased visual motion suppression. The weak suppression was mainly caused by the better direction discrimination of high contrast large stimuli in the remitted MDD group compared to healthy subjects. In contrast to the large stimuli, both MDD and healthy groups performed equally well on the high contrast small stimuli (Fig. 3 of that paper [21]) (see also [32]). These findings leave open whether, in addition to the remitted subjects [21], visual-perceptual deficits can also be observed in acute depressed MDD. This also raises the question whether visual impairment is related to symptom severity in the acute depressed state of MDD (rather than to some other factor like medication as it may exert its impact in remitted subjects).

Employing a similar paradigm as Golomb et al. [21] in acute depressed MDD, we obtained analogous findings, namely weak motion suppression in MDD, thus corroborating the previous findings. However, unlike Golomb et al. [21] who observed the main deficit in large stimuli in their remitted MDD sample, we found that our acute MDD subjects exhibit the most pronounced visual deficit in the small (rather than large) stimuli (Fig. 1). Importantly, this was obtained in both of our acute depressed MDD samples including large and small groups. This strongly suggests that acute depressed MDD subjects exhibit reduced fine-grained differentiation in visual perception.

### From reduced occipital GABA to altered motion suppression—mechanisms

We found significantly lower GABA concentration in hMT+ of acute depressed MDD subjects compared to healthy control subjects (Fig. 3 a). This is in line with the analogous GABA reduction in EVC [6] which we here extend to higher-order visual regions, namely hMT+ (see supplementary discussion for recent controversy about occipital GABA in MDD). How GABA concentrations in lower-order EVC and higher-order hTM+ are related to each other remains subject to future study.

The relevance of occipital GABA is further supported by the relationship of hMT+ GABA concentration to the subjects’ performance in visual motion suppression. Motion SI significantly correlates with GABA concentration (*r* = 0.49, *p* = 0.03) in the HC group; that suggests modulation of fine-grained motion suppression by GABA-ergic inhibition [16]. This relationship is disrupted in MDD. Unlike in healthy subjects, impaired motion SI no longer correlates with reduced hTM+ GABA concentration (*r* = −0.22, *p* = 0.43) in acute MDD subjects. However, we observed that impaired SI is negatively related to symptom severity. This suggests, albeit tentatively, that the disrupted link of reduced hTM+ GABA to motion suppression may be instrumental in bringing forth psychopathological symptoms. The exact mechanisms of this connection remain yet unclear, though.

Suppressive center-surround interactions are a ubiquitous property of visual information processing [33] in many species (for example, human, monkeys, cats, and mouse [16, 34, 35]) including visual motion, orientation [36], and color processing [37]. Various findings in both animals and healthy human subjects [16, 38, 39] clearly show that the balance (interaction) of excitation and inhibition (EIB) mediates visual spatial suppression. These findings are well in line and confirmed by our observations in healthy subjects. Our hierarchical regression analysis demonstrates that the conjoint action of GABA and glutamate exerts the strongest impact on motion suppression, i.e., SI, as distinguished from the effects of GABA and glutamate alone. That, in contrast, is no longer the case in acute MDD, though. Neither GABA and glutamate alone nor their combination relate to the SI in acute MDD anymore. Accordingly, acute MDD subjects may have lost their ability to modulate fine-grained visual perception, i.e., motion suppression, through their EIB which, as we suppose, may be related to reduced GABA and glutamate concentrations.

### Limitations

Some limitations shall be mentioned. It is possible that medication has an influence on the neurochemical levels. We choose only MDD subjects treated with SSRI agents to have a homogenous group and did not observe any changes in our main findings when including medication as co-variate (*Supporting Information*). Rather than reducing GABA level, SSRI have been shown to increase GABA levels [14]; this makes it rather unlikely that reduced GABA in our sample is caused by SSRI. Yet another limiting factor is the rather low subject number for which reason our MRS results must be considered preliminary. To our advantage, we combined 7T MRS with psychophysical testing which allowed us to link occipital GABA to visual perception and psychopathological symptoms. Nevertheless, future larger-scale MRS GABA studies with MDD and psychiatric comparison groups are necessary to support the utility of occipital GABA as potential biomarker of acute MDD.

## Conclusion

We connect biochemical, psychophysical, and psychopathological levels of occipital cortex in acute depressed MDD. We demonstrate reduced GABA in higher-order occipital cortex with its disrupted relation to a specific fine-grained visual impairment in acute MDD which, in turn, relates to symptom severity. Our findings strongly support the assumption of altered GABA in occipital cortex of MDD including its relevance for visual perception and psychopathological symptoms. In conclusion, our findings suggest an important yet unclear role of the occipital cortex in acute MDD including its suitability as candidate biomarker and stimulation treatment target [5] of acute depressed MDD.

## Supplementary information

Supplementary Information

Supplementary Table 1

Supplementary Table 2

Supplementary Table 3

Supplementary Table 4

Supplementary Figure 1

Supplementary Figure 2

Supplementary Figure 3
