# Supplementary material for: Reduction of higher-order occipital GABA and impaired visual perception in acute major depressive disorder
Source: Mol Psychiatry. 2021 Apr 16;26(11):6747–55. doi: 10.1038/s41380-021-01090-5 (PMC8760062; doi:10.1038/s41380-021-01090-5)
Supplement: Supplementary file 1 — Supplementary Information [file 41380_2021_1090_MOESM1_ESM.docx]

**Supplementary material**

**Supplementary method**

**Inclusion and exclusion criteria**

Inclusion criteria of the MDD subjects were: (i) presence of an acute depressive episode and the diagnosis MDD in accordance with the Diagnostic and Statistical Manual of Mental Disorders, Fifth Edition (DSM-V) as (a) established by the assessing psychiatrist, and (b) confirmed with Mini International Neuropsychiatric Interview (M.I.N.I.) [40]; (ii) clinical symptoms of depression as measured by a HAMD-17 score ≥17; (iii) MDD subjects treated with the agent of selective serotonin reuptake inhibitors (SSRI). Given the special scanning in 7T, inclusion criteria of the MDD subjects were: (i) presence of an acute depressive episode and the diagnosis MDD in accordance with the Diagnostic and Statistical Manual of Mental Disorders, Fifth Edition (DSM-V) as (a) established by the assessing psychiatrist, and (b) confirmed with Mini International Neuropsychiatric Interview (M.I.N.I.) [40]; (ii) clinical symptoms of depression as measured by a HAMD-17 score ≥18; (iii) MDD subjects treated with the agent of selective serotonin reuptake inhibitors (SSRI). Exclusion criteria were: (i) any other psychiatric disorder, or a mental disorder caused by a physical illness or substance abuse or a personality disorder; (ii) history of traumatic brain injury, epilepsy or other known organic lesion of the central nervous system; (iii) presence of psychotic symptoms during the depressive episodes; (iv) subjects who were taking benzodiazepines during experiment time as they may affect occipital GABA concentration; and (v) history of endocrine disease or blood, heart, liver, kidney dysfunction, another medical disorder such as diabetes, or pregnancy.

**Detailed procedure for measurement of motion spatial suppression**

Study 1(a large sample of participants) was performed in hospital, we used the portable computer (1920 ×1080 resolution, 144-Hz refresh rate, ROG 3) to conduct the motion suppression experiment in acute depressed MDD patients (*n* = 70) and HC subjects(*n* = 52). The luminance of the display was linearized, gray background was 56 cd/m^2^, and the viewing distance was 47 cm from the screen. A chinrest was used to stabilize head position in these behavioral experiments. Study 2(smaller sample, MDD patients, *n* = 18; HC group, *n* = 20) was performed in the 7T imaging center, visual stimuli were shown on a linearized monitor (1920 ×1080 resolution, 100-Hz refresh rate, Cambridge Research System, UK). The viewing distance was 72 cm from the screen, with the head stabilized by a chinrest. Stimuli were drawn against a gray (56 cd/m^2^) background.

A schematic of the stimuli and trial sequences is shown in Supplementary Figure 1(Psychophysical Task). The stimulus was a vertical drifting sinusoidal grating (contrast, 50%(high contrast); spatial frequency, 1 cycle/o; speed, 4°/s) of either small (diameter of 2°) or large (diameter of 10°) size. The edge of the grating was blurred with a raised cosine function (width, 0.3°). A cross was presented in the center of the screen at the beginning of each trial for 500ms, and participants were instructed to fixate at the cross and to keep fixating at the cross throughout the trial. In each trial, a grating of either large or small size was randomly presented at the center of the screen. The grating drifted either leftward or rightward, and participants were asked to judge the perceived moving direction by a key press. An auditory feedback was provided in each wrong response.

**Detailed procedure for locating the hMT+ VOI**

The hMT+ VOIs were placed in the ventrolateral occipital lobe, which was based on anatomical landmarks [16, 28]. In these hMT+ VOIs, we did not distinguish between the middle temporal (MT) and medial superior temporal (MST) areas [16], both of which are motion selective [41]. For 10 subjects, we, using fMRI, also functionally identified hMT+ as a check on the placement of the VOI (as in the method in ref [16]). We did not scan the functional localizer for all MDD patients in order to reduce experimental load and time for the acute depressed subjects. In addition, we only used the left hMT+ as the target region to scan, which was motivated by studies showing that left hMT+ is more related to visual perceptual effects [42].

**Detailed procedure for estimating the metabolite concentrations in hMT+ VOIs**

First, we used the non-suppressed water spectra to perform eddy current correction and frequency/phase correction. Second, we checked the quality of each FID (16 averages) visually and removed those with obviously poor quality. Third, the absolute concentrations of each metabolite were quantitatively estimated via the Water-Scaling method. For partial-volume correction, the tissue water content was computed as follows [43]:

Tissue water content = *f*_gm_*0.78 + *f*_wm_*0.65 + *f*_csf_*0.97 (2)

where *f*_gm_, *f*_wm_, and *f*_csf_ were the GM/WM/CSF volume fraction in MRS VOI and we used FAST (FMRI ’s automated segmentation tool, part of the FSL toolbox) [44] to segment the three tissue compartments from the T1-weighted structural brain images. For water T2 correction, we set water T2 as 47ms [45].

**Supplementary Results**

**Biochemical findings - Other metabolite concentrations in hMT+**

The levels of all metabolites obtained by LCModel analysis in hMT+ VOI for both the MDD and HC groups are given in in Supplementary Table 3. The levels of multiple metabolites, including Glu (*p* = 0.02), NAA (*p* = 0.02), GABA (*p* < 0.05), mI (*p* = 0.03), GSH (*p* = 0.03) and PCr (*p* = 0.02) were significantly lower in participants with MDD than in HCs (all *p* values were correlated for multiple comparisons with FDR).

We calculated the cross-correlation matrix among all metabolites including GABA and glutamate (see Fig. S2); this yielded the expected results like the well-known correlation of Glu with NAA. We can also see that the cross-correlation structure was more or less the same in both HC and MDD with the exception of GABA that exhibited a different, i.e., more distant or none at all, relationship with the other metabolites in MDD (when compared to HC).

Finally, we checked for the confounding impact of voxel tissue composition. Mean MRS Voxel tissue compositions (gray matter, white matter and CSF) are shown in Supplementary Table 3 for MDD and HC groups. No significant differences in voxel composition were found between groups. This makes it rather unlikely that tissue composition accounts for GABA concentration differences in our relatively young sample of MDD subjects (22.8 $\pm$ 4.1 years), which were similar in age to a group of first-episode psychosis subjects (22.3 $\pm$ 4.4 years) which showed similar lack of difference in tissue composition to healthy controls; [46]).

**Supplementary discussion**

**GABA and occipital cortex in MDD – A controversial relationship?**

Several studies report reduced GABA and glutamatergic metabolism levels of MDD patients in various regions of the brain including anterior cingulate cortex (ACC), the dorsolateral, dorsomedial, and dorsoventral prefrontal cortex, the amygdala, and the hippocampus [9, 10, 47–49]. Additionally, several studies also observed reduced GABA (and abnormal glutamate) concentrations in occipital cortex, i.e., early visual cortex (EVC) of MDD patients [6]. However, a recent study and meta-analysis could not confirm these earlier findings citing various methodological problems including the limited GABA resolution in 3T MRS [8]. This puts into doubt whether occipital GABA reduction is valid and does indeed occur in MDD.

To obtain more valid spectra, we used ultra-high field (7T) ^1^H-MRS technology to obtain higher and less noisy GABA signals. MRS at 7T allows to accurately estimate levels of compounds which have small and/or overlapping signals in brain spectra. For example, the ability to resolve glutamate (Glu) from glutamine (Gln) [50, 51], as well as to measure glutathione (GSH) and GABA with greater accuracy than in 3T [52, 53]. Moreover, rather than focusing on early visual cortex, we targeted higher-order occipital cortex, that is, hTM+. Finally, we combined MRS with a psychophysical perception task that, as well known, is mediated by hTM+ [15, 16]. Together, these methodological steps allowed us to ascertain the validity of GABA and to further probe it by relating it to corresponding visual impairment on the psychophysical level of visual perception.

**Supporting Information**

We tested for this and did not observe significant change in our results as it is now explicitly mentioned in our results. Moreover, it shall be mentioned that all MDD subjects were on the same serotoninergic drug and dosages. However, we admit that the main limitation of the present study was the possible confounding effects of medication. Indeed, almost all of the acute MDD patients in our sample were taking medications, including mood stabilizers, antipsychotics, antidepressants, and benzodiazepines (see supplementary table 2), which may possibly influence results. Following recent suggestions and standards, we examined the potential impact of the psychotropic medication load—the number and dosage of different medications, we then used the codes 0, 1, 2, and 3 to indicate no medication, and dose-equivalents below, equal, or above the mean effective daily dose, respectively [54]. We generated a composite measure of the medication load by summing all individual medication codes for each category and each individual MDD patient. We investigated the potential impact of medications on MRS data by correlating the resulting pharmacological load with all molecules tested. The medication load did not correlate with this measure in antidepressants, mood stabilizers and benzodiazepines. The antipsychotic load also not correlate with GABA concentration (*r* = -0.28, *p* = 0.29), while it significantly correlated with Glu concentration (*r* = -0.66, *p* = 0.005).

Then, to further control for an eventual effect of pharmacotherapy on all molecules tested, we compared this variable by using a *t* test for each medication class (mood stabilizers, benzodiazepines, and antipsychotics), between those patients who were in treatment with the respective drug and those who were not. We found no differences between patients who were in treatment with mood stabilizers (*n* = 6) and patients who were not (*n* = 10) (*p* > 0.05), between patients who were in treatment with benzodiazepines (*n* = 8) and patients who were not (*n* = 8) (*p* > 0.05). There was no difference between patients who were in treatment with antipsychotics (*n* = 10) and patients who were not (*n* = 6) for GABA concentration (*p* = 0.68), while there was significant difference between patients who were in treatment with antipsychotics (*n* = 10) and patients who were not (*n* = 6) for Glu concentration (*p* = 0.007).

**Supplementary references**

40. Sheehan D V, Lecrubier Y, Sheehan KH, Amorim P, Janavs J, Weiller E, et al. The Mini-International Neuropsychiatric Interview (M.I.N.I.): the development and validation of a structured diagnostic psychiatric interview for DSM-IV and ICD-10. J Clin Psychiatry. 1998;59 Suppl 2:22-33;quiz 34-57.

41. Huk AC, Dougherty RF, Heeger DJ. Retinotopy and Functional Subdivision of Human Areas MT and MST. J Neurosci. 2002;22:7195–7205.

42. Tadin D, Silvanto J, Pascual-Leone A, Battelli L. Improved Motion Perception and Impaired Spatial Suppression following Disruption of Cortical Area MT/V5. J Neurosci. 2011;31:1279–1283.

43. Ernst T, Kreis R, Ross BD. Absolute Quantitation of Water and Metabolites in the Human Brain. I. Compartments and Water. J Magn Reson Ser B. 1993;102:1–8.

44. Zhang Y, Brady M, Smith S. Segmentation of brain MR images through a hidden Markov random field model and the expectation-maximization algorithm. IEEE Trans Med Imaging. 2001;20:45–57.

45. Marjańska M, Auerbach EJ, Valabrègue R, Van de Moortele PF, Adriany G, Garwood M. Localized 1H NMR spectroscopy in different regions of human brain in vivo at 7T: T 2 relaxation times and concentrations of cerebral metabolites. NMR Biomed. 2012;25:332–339.

46. Wang AM, Pradhan S, Coughlin JM, Trivedi A, Dubois SL, Crawford JL, et al. Assessing Brain Metabolism with 7-T Proton Magnetic Resonance Spectroscopy in Patients with First-Episode Psychosis. JAMA Psychiatry. 2019;76:314–323.

47. Hasler G, Northoff G. Discovering imaging endophenotypes for major depression. Mol Psychiatry. 2011;16:604–619.

48. Michael N, Erfurth A, Ohrmann P, Arolt V, Heindel W, Pfleiderer B. Neurotrophic Effects of Electroconvulsive Therapy: A Proton Magnetic Resonance Study of the Left Amygdalar Region in Patients with Treatment-Resistant Depression. Neuropsychopharmacology. 2003;28:720–725.

49. Pittaluga A, Raiteri L, Longordo F, Luccini E, Barbiero VS, Racagni G, et al. Antidepressant treatments and function of glutamate ionotropic receptors mediating amine release in hippocampus. Neuropharmacology. 2007;53:27–36.

50. Ende G. Proton Magnetic Resonance Spectroscopy: Relevance of Glutamate and GABA to Neuropsychology. Neuropsychol Rev. 2015;25:315–325.

51. Tkáč I, Öz G, Adriany G, Uǧurbil K, Gruetter R. In vivo 1H NMR spectroscopy of the human brain at high magnetic fields: Metabolite quantification at 4T vs. 7T. Magn Reson Med. 2009;62:868–879.

52. Mekle R, Mlynárik V, Gambarota G, Hergt M, Krueger G, Gruetter R. MR spectroscopy of the human brain with enhanced signal intensity at ultrashort echo times on a clinical platform at 3T and 7T. Magn Reson Med. 2009;61:1279–1285.

53. Pradhan S, Bonekamp S, Gillen JS, Rowland LM, Wijtenburg SA, Edden RAE, et al. Comparison of single voxel brain MRS AT 3T and 7T using 32-channel head coils. Magn Reson Imaging. 2015;33:1013–1018.

54. Davis JM, Chen N. Dose Response and Dose Equivalence of Antipsychotics. J Clin Psychopharmacol. 2004;24:192–208.

**Supplementary figure legends**

**Supplementary Figure 1 Illustration stimuli and task**

**a** Fixation: Participants should fixate at the cross and keep fixating during the trial. **b** Stimuli: 2 kinds of motive direction, left or right; 2 kinds of sizes, small (diameter of 2^o^) or large (diameter of 10^o^). **c** Task: Judge the motive direction of grating (no time limitation). Sound of feedback for wrong choose.

**Supplementary Figure 2 The cross correlation structure in the level of all molecules tested**

Cross correlation structure showing the correlation coefficients between all metabolite concentration in MDD (upper, *n* = 16) and HC (below, *n* = 20) hMT+ area

**Supplementary Figure 3 GABA or Glu moderation of SI effects on HAMD**

Conceptual and statistical depiction of the effects tested, ie, the main effect of each of the 2 factors and their iteration. We used the independent variable(X)-SI, moderation variable (M)-Glu/or GABA, and the interactive term of SI and Glu/or GABA to explain the dependent variable(Y) of HAMD
