# Supplementary material for: Reduction of higher-order occipital GABA and impaired visual perception in acute major depressive disorder
Source: Mol Psychiatry. 2021 Apr 16;26(11):6747–55. doi: 10.1038/s41380-021-01090-5 (PMC8760062; doi:10.1038/s41380-021-01090-5)
Supplement: Supplementary file 2 — Supplementary Table 1 [file 41380_2021_1090_MOESM2_ESM.docx]

**Supplementary Table 1**

**Table S1.** Demographics and participant psychophysics data and patient clinical data in a large sample.

| Variables | MDD patients  (N = 70) | Healthy controls  (N = 52) | *p* value |
| --- | --- | --- | --- |
| Gender (M/F) | 12/58 | 18/34 | **0.033** |
| Age, years (SD) | 23.4 (5.11) | 24.5 (4.0) | 0.221 |
| Education, years (SD) | 14.7 (1.9) | 16.4 (1.3) | **< 0.001** |
| Suppression index (SD) | 0.15 (0.08) | 0.16 (0.09) | 0.473 |
| HAMD-17 scores (SD) | 20.8 (3.6) | - | - |
| Treatment, n (%) |  |  |  |
| Antidepressants | 64 (91.4) | - | - |
| SSRI | 64 (91.4) | - | - |
| Combination | 8 (11.4) | - | - |
| No antidepressants | 6 (8.6) | - | - |
| Antipsychotics | 34 (48.6) | - | - |
| Benzodiazepines | 40 (57.1) | - | - |
| Mood stabilizers | 8 (11.4) | - | - |

Bold font indicates *p* < 0.05.
