# Supplementary material for: Reduction of higher-order occipital GABA and impaired visual perception in acute major depressive disorder
Source: Mol Psychiatry. 2021 Apr 16;26(11):6747–55. doi: 10.1038/s41380-021-01090-5 (PMC8760062; doi:10.1038/s41380-021-01090-5)
Supplement: Supplementary file 3 — Supplementary Table 2 [file 41380_2021_1090_MOESM3_ESM.docx]

**Supplementary Table 2**

**Table S2.** Demographics and participant psychophysics data and patient clinical data in smaller sample

| Variables | MDD patients  (N = 18) | Healthy controls  (N = 20) | *p* value |
| --- | --- | --- | --- |
| Gender (M/F) | 8/10 | 10/10 | 0.505 |
| Age, years (SD) | 22.8 (4.1) | 23.4 (2.1) | 0.373 |
| Education, years (SD) | 15.2 (1.5) | 16.2 (1.4) | 0.054 |
| Suppression index (SD) | 0.05 (0.09)^*^ | 0.10 (0.06) | **0.007** |
| HAMD-17 scores (SD) | 23.8 (3.6) | - | - |
| Treatment, n (%) |  |  |  |
| Antidepressants | 17 (94.4) | - | - |
| SSRI | 17 (94.4) | - | - |
| Combination | 1 (5.6) | - | - |
| No antidepressants | 1 (5.6) | - | - |
| Antipsychotics | 12 (66.7) | - | - |
| Benzodiazepines | 9 (50) | - | - |
| Mood stabilizers | 6 (33.3) | - | - |

^*^ One patient was unable to complete the psychophysics task, so N=17. Bold font indicates *p* < 0.05.
