# Supplementary material for: Reduction of higher-order occipital GABA and impaired visual perception in acute major depressive disorder
Source: Mol Psychiatry. 2021 Apr 16;26(11):6747–55. doi: 10.1038/s41380-021-01090-5 (PMC8760062; doi:10.1038/s41380-021-01090-5)
Supplement: Supplementary file 4 — Supplementary Table 3 [file 41380_2021_1090_MOESM4_ESM.docx]

**Supplementary Table 3**

**Table S3.** Metabolite concentrations and CSF/GM/WM fraction

|  | MDD | | |  | HC | | | Statistic |
| --- | --- | --- | --- | --- | --- | --- | --- | --- |
|  | Mean | SD | N |  | Mean | SD | N | *p* value |
| Cr | 2.66 | 0.46 | 13 |  | 2.90 | 0.73 | 15 | 0.3868 |
| GABA | 1.76 | 0.37 | 16 |  | 2.02 | 0.29 | 20 | **0.0495** |
| Glu | 6.89 | 0.91 | 16 |  | 8.07 | 1.30 | 20 | **0.0171** |
| Gln | 1.15 | 0.22 | 11 |  | 1.30 | 0.39 | 12 | 0.3740 |
| GSH | 1.59 | 0.27 | 16 |  | 1.87 | 0.34 | 20 | **0.0343** |
| mI | 5.15 | 0.85 | 16 |  | 5.93 | 0.94 | 20 | **0.0343** |
| NAA | 10.79 | 1.80 | 16 |  | 12.81 | 1.82 | 20 | **0.0171** |
| PCr | 4.28 | 0.72 | 16 |  | 5.11 | 0.82 | 20 | **0.0171** |
| Tau | 1.22 | 0.32 | 16 |  | 1.39 | 0.47 | 16 | 0.3244 |
| CSF | 0.02 | 0.01 | 16 |  | 0.03 | 0.02 | 20 | 0.1916 |
| GM | 0.39 | 0.05 | 16 |  | 0.39 | 0.07 | 20 | 0.9310 |
| WM | 0.59 | 0.06 | 16 |  | 0.58 | 0.08 | 20 | 0.6651 |

N: number of samples, Cr: creatine, GABA: γ-aminobutyric acid, Gln: glutamine, Glu: glutamate, GSH: glutathione, mI: myo-inositol, NAA: N-asetylaspartate, PCr: phosphocreatine, Tau: taurine. *p* value: with FDR correction. *p* values < 0.05 showed with bold.
