# Supplementary material for: Reduction of higher-order occipital GABA and impaired visual perception in acute major depressive disorder
Source: Mol Psychiatry. 2021 Apr 16;26(11):6747–55. doi: 10.1038/s41380-021-01090-5 (PMC8760062; doi:10.1038/s41380-021-01090-5)
Supplement: Supplementary file 5 — Supplementary Table 4 [file 41380_2021_1090_MOESM5_ESM.docx]

**Supplementary Table 4**

**Table S4.** The results of the moderation model analysis

| X (independent variable) | Y (dependent variable) | M (moderator variable) | *p* value of the model | *p* value of the interactive term |
| --- | --- | --- | --- | --- |
| SI | HAMD | GABA | 0.284 | 0.618 |
| SI | HAMD | Glu | 0.054 | 0.071 |
