# Supplementary figures and images for: Reduction of higher-order occipital GABA and impaired visual perception in acute major depressive disorder
Source: Mol Psychiatry. 2021 Apr 16;26(11):6747–55. doi: 10.1038/s41380-021-01090-5 (PMC8760062; doi:10.1038/s41380-021-01090-5)

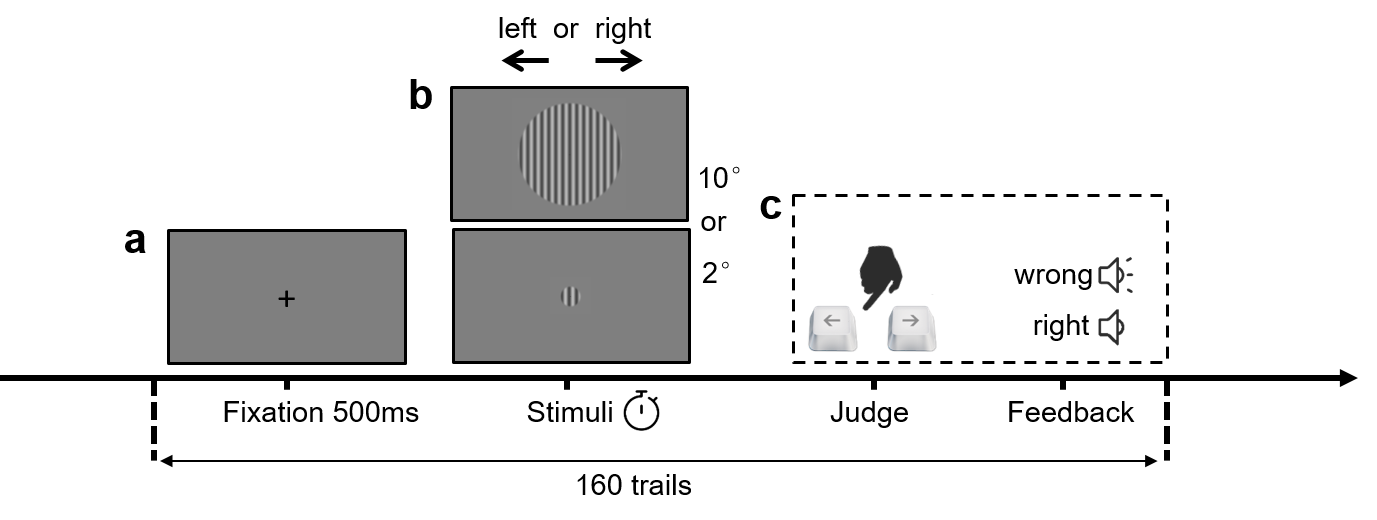

Supplement: Supplementary file 6 — Supplementary Figure 1 [file 41380_2021_1090_MOESM6_ESM.tif]

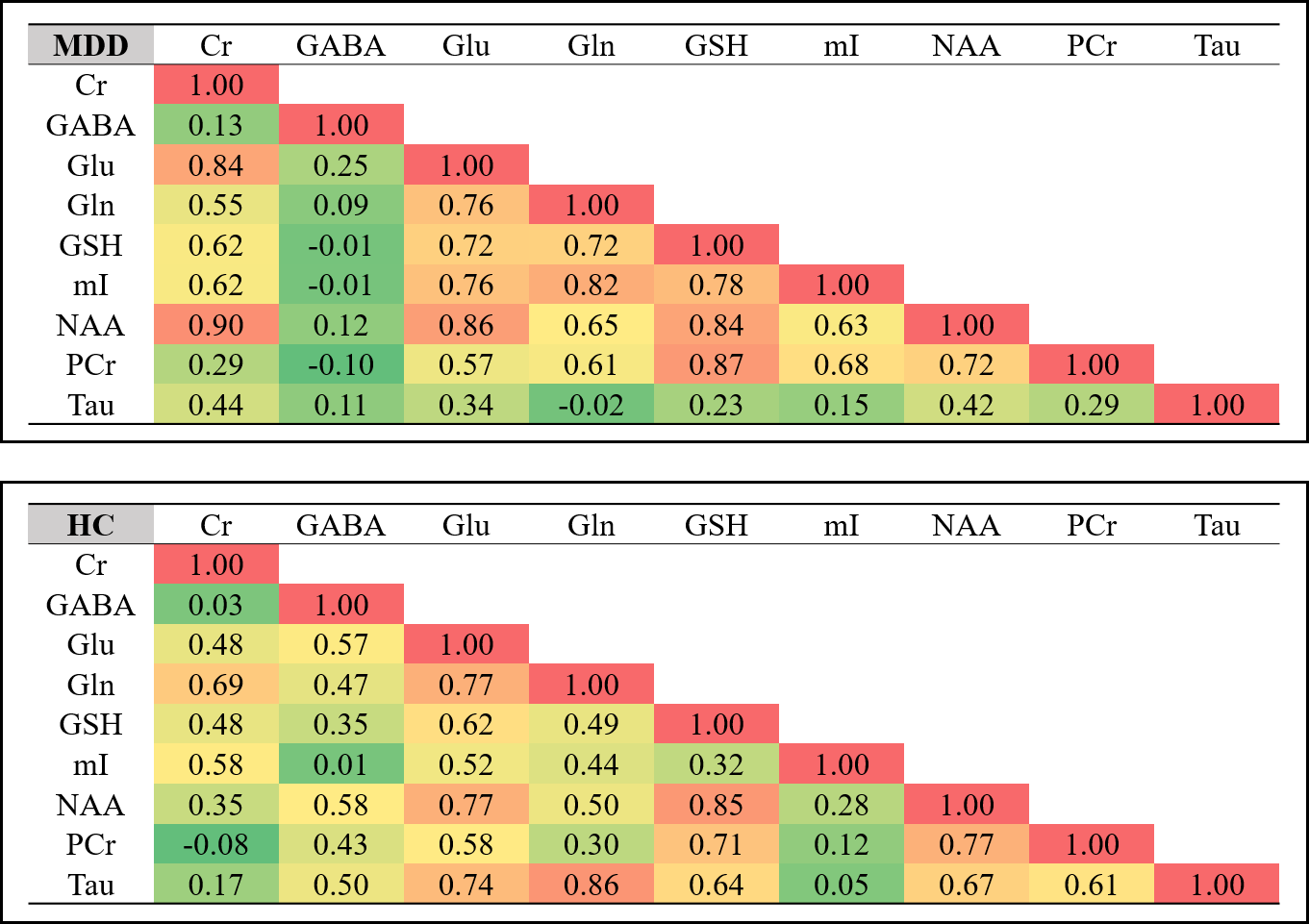

Supplement: Supplementary file 7 — Supplementary Figure 2 [file 41380_2021_1090_MOESM7_ESM.tif]

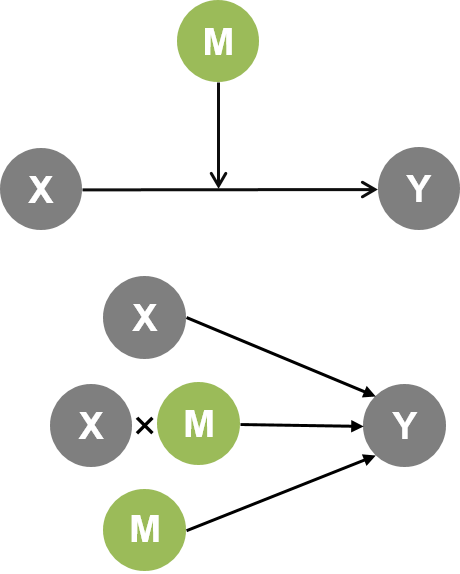

Supplement: Supplementary file 8 — Supplementary Figure 3 [file 41380_2021_1090_MOESM8_ESM.tif]
